# Functional impairment is associated with an increased risk of mortality in patients on chronic hemodialysis

**DOI:** 10.1186/s12882-016-0302-y

**Published:** 2016-07-08

**Authors:** Maurizio Bossola, Enrico Di Stasio, Manuela Antocicco, Gilda Pepe, Luigi Tazza, Giuseppe Zuccalà, Alice Laudisio

**Affiliations:** Department of Surgery, Hemodialysis Service, Catholic University of the Sacred Hearth, Largo A. Gemelli, 8 – 00168 Rome, Italy; Department of Clinical Chemistry, Catholic University of the Sacred Hearth, Largo A. Gemelli, 8 – 00168 Rome, Italy; Department of Gerontology, Geriatrics and Psychiatry, Catholic University of the Sacred Hearth, Largo A. Gemelli, 8 – 00168 Rome, Italy; Department of Medicine, Unit of Geriatrics, Campus Bio-Medico di Roma University, Rome, Italy

**Keywords:** End-stage renal disease, Hemodialysis, ADLs, IADLs impairment, Depression, Mortality

## Abstract

**Background:**

Functional impairment is associated with adverse outcomes in older people, as well as in patients on chronic hemodialysis. The aim of the present study was to determine the characteristics associated with functional impairment in chronic hemodialysis, and to evaluate if functional impairment represents a risk factor for reduced survival in chronic hemodialysis.

**Methods:**

All 132 chronic hemodialysis referring to the Hemodialysis Service of the Catholic University, Rome, Italy between November 2007 and May 2015 were included. All patients underwent comprehensive geriatric assessment; functional ability was estimated using two questionnaires exploring independency in bathing, dressing, toileting, transferring, continence, feeding (ADLs), and independency in using the telephone, shopping, food preparation, housekeeping, laundering, traveling, taking medications, and handling finances (IADLs). Functional impairment was diagnosed in presence of dependence in one or more ADLs/IADLs. Mood was assessed using the 30-item Geriatric Depression Scale. Logistic regression was used to evaluate factors associated with functional impairment. The association between functional impairment and survival was assessed by Cox regression.

**Results:**

ADLs impairment was present in 34 (26 %) participants, while IADLs impairment was detected in 64 (48 %) subjects. After a follow up of 90 months, 55 (42 %) patients died. In logistic regression, depressive symptoms were associated with ADLs and IADLs impairment (OR 1.12; 95 % CI = 1.02–1.23; OR 1.16; 95 % CI = 1.02–1.33; respectively). In Cox regression, ADLs impairment was associated with mortality (HR 2.47; 95 % CI–1.07–5.67) while IADLs impairment was not associated with reduced survival (HR .80; 95 % CI–.36–1.76).

**Conclusions:**

Functional impairment is associated with depressive symptoms; also, impairment in the ADLs represents a risk factor of reduced survival in chronic hemodialysis. These associations and their potential implication should be assessed in dedicated studies.

## Background

Functional ability refers to a person’s ability to perform basic tasks without assistance.

Such activities may be described as “fundamental”activities of daily living (ADLs) or instrumental activities of daily living (IADLs) [1–3]. The ADLs explore independency in bathing, dressing, toileting, transferring, continence, and feeding, while the IADLs rate independency in using the telephone, shopping, food preparation, housekeeping, laundering, traveling, taking medications, and handling finances [1–3].

Reduced or absent functional ability is recognized as a contributor to recurrent hospitalization and increased mortality in the general population and in patients with chronic diseases [4, 5].

With the aging of populations, an increasing number of elderly patients with end-stage renal disease is starting dialysis. In addition, chronic hemodialysis patients develop progressive functional impairment after the initiation of replacement therapy [6]. Accordingly, impairment in the ADLs, IADLs, or both affect up to 50 % of elderly patients on chronic hemodialysis [7–9].

Definition of functional assessment is useful to define the prognosis and the knowledge of factors associated with functional impairment may help identify “high risk patients” in whom therapeutic interventions might yield a beneficial cost effectiveness profile.

Unfortunately, little is known about the characteristics of disabled subjects on chronic hemodialysis as well as about their prognosis [10, 11]. Thus, with the present study, we aimed to determine the demographic, clinical and laboratory variables associated with ADLs and/or IADLs impairment in patients on chronic hemodialysis, and to evaluate if ADL and/or IADL impairment represents a risk factor for increased mortality in such patients.

## Methods

### Study design and participants

One hundred forty nine patients on chronic thrice weekly hemodialysis for at least 6 months at the Hemodialysis Unit of the Catholic University, Rome, Italy, between November 2007 and May 2015 were screened for inclusion in the study. Of these, we excluded 17 patients because of: inability to answer to the questionnaires because of deafness or reading problems (7 patients), diagnosis of dementia (3 patients), instability of clinical conditions requiring hospitalization (2 patients), and diagnosis of liver insufficiency or active cancer (5 patients). Therefore, complete data for the present study were available for 132 participants. The only sociodemographic characteristics in which excluded participants differed from those included in this study were older age (71 ± 8 years vs 62 ± 15 years; *P* = .023), and a lower dialytic blood flow (270 ± 31 mL/min vs 285 ± 25 mL/min; *P* = .029).

The study protocol was approved by the Ethics Committee of the Catholic University of Rome (number: P/607/CE; year: September/2011) and all participants provided written informed consent. The study has been performed in accordance with the ethical standards laid down in the 1964 Declaration of Helsinki. The experiments comply with the current laws of Italy.

### Functional ability

Functional ability was estimated using the Katz’ ADLs [2], and the Lawton and Brody scale for IADLs [3]. These scales are used to assess functional independency for clinical and epidemiological purposes. The ADLs scale defines the independency in bathing, dressing, toileting, transferring, continence, and feeding. A point is given for independence in each area, up to a maximum score of six for independence in all the domains [2]. The IADLs scale explores independency in using the telephone, shopping, food preparation, housekeeping, laundering, traveling, taking medications, and handling finances. A point is given for independence in each area, with a total score of eight for independence in all domains [3]. Impairment in the ADLs was defined as need of assistance for performing one or more ADLs. Impairment in IADL was stated as need of assistance for performing one or more ADLs. Also, increasing levels of dependency were examined, considering ADL score cutoffs < 2, between 2 and 4, and > 4, as well as an IADL score < 3, between 3 and 5, and >5.

### Covariates

All patients were receiving conventional 4-hour hemodialysis, three times a week. The blood flow ranged from 250 to 300 mL/min with a dialysis rate flow of 500 mL/min. All patients were treated with high-permeability membranes. Membranes were not reused. Body Mass Index was calculated as weight (Kg) divided by height squared (m^2^). Depressive symptoms were evaluated using the validated Italian version of the 30-item Geriatric Depression Scale (GDS) [12]. Comorbidity was assessed by the Charlson Comorbidity Index [13]. Cognitive function was evaluated using the Mini Mental State Examination (MMSE) that was administered in the middle of the week [14, 15]. Time of recovery after hemodialysis was also recorded asking to the patients “How long does it take you to recover from a dialysis session?” [16]. Blood samples were obtained after overnight fasting immediately before the hemodialysis session. Laboratory parameters were measured at the Department of Laboratory Medicine, Catholic University of Rome.

### Statistical analyses

Statistical analyses were performed using SPSS for Mac 20.0. Differences were considered significant at the P < .050 level. The covariates to be entered into analyses, besides their descriptive value, were chosen as explanatory according to available reviews and meta-analyses from the electronic databases of PubMed (MEDLINE) and Cochrane Library, and based upon their efficiency and economicity. Data of continuous variables are presented as mean values ± standard deviation (SD). Medians and inter-quartile ranges were provided for non-normally distributed variables. Analysis of variance (ANOVA) for normally distributed variables was performed according to impairment in the ADLs; otherwise, the nonparametric Mann–Whitney *U* test was adopted. The two-tailed Fisher exact test was used for dichotomous variables. Pearson’s correlation analysis was performed to assess the correlation of serum creatinine levels, serum albumin levels and Body Mass Index.

Multivariable logistic regression analysis was adopted to estimate the association of ADLs and IADLs impairment with age, sex, and all those variables, which differed significantly (P < .05) in univariate analyses. Also, multivariable logistic regression was used to assess the association between depression (i.e. 30 item GDS score >11) and increasing levels of functional impairment. Survival analysis was performed using the Kaplan-Meyer method. Univariate analysis was performed in order to examine the relationship between potential predictor variables and death. Variables associated to mortality were included as potential confounders in a multivariable Cox proportional hazard regression analysis. In addition, to avoid any possible inflated association of patients who die early (i.e. first six months of follow-up) the same Cox analysis was ran after the exclusion of these subjects. The area under the ROC curve was adopted to compare if and how the models perform.

As malnutrition and low muscle mass are acknowledged risk factors for functional impairment and mortality, and creatinine levels are known to reflect both conditions, the dependency of the association between functional ability and survival upon creatinine levels was assessed by the analysis of the interaction term “ADLs*serum creatinine” using the multivariable Cox model. In addition, Cox regression analysis was also adopted to evaluate the adjusted association between increasing levels of functional impairment (ADL score: 0–2, 2–4, and ≥ 4; IADL score 0–3, 3–5, and ≥ 5) and survival.

## Results

Impairment in the ADLs was recorded in 34/132 (26 %) patients, while impairment in the IADLs was detected in 64/132 (48 %) patients. Impairment in five ADLs was found in 1 (0.7 %) subject, in four ADLs in 4 (3 %) participants, and in three ADLs in 11 (8.1) subjects; impairment in two ADLs was established in 5 (3.7 %) patients, and in one ADL in 13 (9.6 %) participants.

In particular, impairment in the ability of bathing was present in 1 patient, in dressing in 19 subjects, in toileting in 18 participants, in the ability of transferring in 5 patients, in continence in 34 subjects, and impaired feeding in 1 participant.

Also, an impairment in all IADLs was found in 1 (0.7 %) patient; impairment in seven IADLs was detected in 5 (3.7 %) subjects, in six IADLs in 9 (6.7 %) participants, in five IADLs in 14 (10.4 %) subjects, in four IADLs in 9 (6.7 %) patients, in three IADLs in 9 (6.7 %) subjects, in two IADLs in 13 (9.6 %) subjects, and in one IADL in 4 (3 %) subjects.

Eventually, impairment in the ability of using the telephone was present in 1 patient, impairment in the ability of shopping in 43, impairment in the ability of food preparation in 45, impairment in the ability of housekeeping in 47, impairment in the ability of laundering in 45, impairment in the ability of traveling in 35, impairment in the ability of taking medications in 28, and impairment in the ability of handling finances in 21.

After a follow-up of 90 months, 55 (42 %) patients died (of those, 7 subjects died within the first six months), while 11/132 (8 %) patients were transplanted. The median ADL and IADL scores were lower in participants who died as compared with survivors (ADL: 4, 3–5 vs 5, 4–6; *P* < .0001; IADL 4, 3–8 vs 7, 6–8; *P* < .001).

### Cross sectional analyses

The characteristics of patients according to the presence of ADLs impairment are depicted in Table 1. Patients with ADL impairment had a higher GDS score as compared with patients without ADL impairment. In addition, they showed lower creatinine serum levels.

**Table 1:** Characteristics of 132 participants according to the presence of impairment in the Activities of Daily Living (ADLs; impairment was defined as an ADLs score <6). (Study center: Catholic University, Rome, 2007–2015)

	Patients with ADLs impairment (*n* = 34) *n* (%), mean ± SD, or median and interquartiles	Patients without ADLs impairment (*n* = 98) *n* (%), mean ± SD, or median and interquartiles	*P*
Age (years)	71 ± 11	61 ± 15	<.01
Sex (female)	14 (41 %)	35 (36 %)	.68
Dialytic age (months)	36 (12–84)	36 (12–83)	.79
Body Mass Index (Kg/m^2^)	25.6 ± 4.7	24.5 ± 4.4	.22
Charlson comorbidity index score	4 (2–5)	2 (1–3)	<.01
Diabetes	15 (44 %)	26 (27 %)	.08
Geriatric Depression Scale	15 ± 6	9 ± 6	<.01
Mini Mental State Examination	22 ± 4	24 ± 3	<.01
Kt/V	1.2 ± 0.3	1.3 ± 0.2	.07
Dialytic blood flow (mL/min)	284 ± 27	284 ± 25	.92
Time of recovery after hemodialysis (min)	210 (60–720)	180 (60–360)	.28
Hemoglobin (g/dL)	11.2 ± 1.2	11.0 ± 1.5	.46
Serum creatinine (mg/dL)	8.5 ± 2.2	10.3 ± 3.0	<.01
Serum albumin (g/dL)	3.8 ± 0.3	4.0 ± 0.3	<.01
Serum calcium (mg/dL)	8.7 ± 0.6	9.0 ± 0.6	.11
Serum phosphorus (mg/dL)	5.6 ± 1.6	5.3 ± 1.8	.49
Serum PTH (mg/dL)	245 (110–350)	278 (146–417)	.21
Serum bicarbonate (mEq/L)	23.5 ± 1.9	24.0 ± 1.8	.20

The characteristics of patients according to the presence of IADLs impairment are shown in Table 2. Patients with IADL impairment had a higher GDS score and lower creatinine serum levels than patients without IADL impairment.

**Table 2:** Characteristics of 132 participants according to the presence of impairment in the Instrumental Activities of Daily Living (IADLs; defined as and IADLs score < 8). (Study center: Catholic University, Rome, 2007–2015)

	Patients with IADLs impairment (*n* = 64) *n* (%), mean ± SD, or median and interquartiles	Patients without IADLs impairment (*n* = 68) *n* (%), mean ± SD, or median and interquartiles	*P*
Age (years)	70 ± 11	57 ± 15	<.01
Sex (female)	29 (45 %)	20 (29 %)	.07
Dialytic age (months)	40 (12–88)	36 (12–75)	.66
Body Mass Index (Kg/m^2^)	24.9 ± 4.4	24.7 ± 4.6	.82
Charlson comorbidity index score	3 (2–4)	2 (1–3)	<.01
Geriatric Depression Scale	14 ± 6	7 ± 5	<.01
Mini Mental State Examination	22 ± 4	24 ± 3	<.01
Kt/V	1.3 ± 0.2	1.2 ± 0.2	.67
Dialytic blood flow (mL/min)	281 ± 27	288 ± 24	.12
Time of recovery after hemodialysis (min)	210 (112–480)	120 (30–300)	.02
Hemoglobin (g/dL)	10.9 ± 1.8	11.3 ± 1.1	.17
Serum creatinine (mg/dL)	8.7 ± 2.2	10.8 ± 3.1	<.01
Serum albumin (g/dL)	3.8 ± 0.3	4.0 ± 0.2	<.01
Serum calcium (mg/dL)	8.9 ± 0.6	9.0 ± 0.6	.30
Serum phosphorus (mg/dL)	5.0 ± 1.5	5.7 ± 1.9	.06
Serum PTH (mg/dL)	265 (123–429)	260 (143─361)	.90

According to logistic regression analysis, ADLs impairment was associated with depressive symptoms expressed by the 20-item GDS in crude model (OR 1.19; 95 % CI = 1.09–1.29), adjusting for age and sex (OR 1.16; 95 % CI = 1.06–1.27) and in the fully adjusted model (OR 1.12; 95 % CI = 1.02–1.23; Table 3), adjusted for those variables which showed significant differences in univariate analyses. Also, IADLs impairment was associated with the GDS score in crude model (OR 1.24; 95 % CI–1.14–1.34), adjusting for age and sex (OR 1.20; 95 % CI = 1.10–1.31) and in the fully adjusted model (OR 1.16; 95 % CI = 1.02–1.33; Table 3), adjusted for those variables which showed significant differences in univariate analyses.

**Table 3:** Association (OR coefficients, and 95 % confidence intervals, CI) of functional impairment with the variables of interest according to logistic multivariable regression model. All the covariates were entered simultaneously into the regression model. (Study center: Catholic University, Rome, 2007–2015)

	ADLs impairment		IADLs impairment	
	OR	95 % CI	OR	95 % CI
Age (years)	1.05	.98–1.13	1.01	.95–1.07
Sex (female)	.92	.28–3.05	2.77	.69–11.05
Charlson comorbidity index score	1.28	.92–1.78	1.26	.86–1.85
Mini Mental State Examination	.97	.83–1.13	.78	.58–1.06
Serum creatinine (mg/dL)	.85	.66–1.10	.78	.57–1.06
Serum albumin (g/dL)	.91	.15–5.66	.89	.08–9.91
Time of recovery after hemodialysis (min)			1.21	.72–2.05
Geriatric Depression Scale	1.12	1.02–1.23	1.16	1.02–1.33

Eventually, depression was not associated with increasing level of ADLs impairment (P for trend–.14).

### Prospective analyses

Patients who died, as compared with survivors, had an higher GDS score (13 ± 5 vs 8 ± 6; *P* = <.01), lower creatinine serum levels (8.6 ± 2.2 vs 10.7 ± 3.0 mg/dL; P < .01), and an higher prevalence of functional impairment both in the ADLs (28, 51 vs 6, 8 %; *P* < .01) and IADLs (37, 67 vs 27, 35 %; *P* < .01).

In Kaplan Meier analysis the log rank test indicated that survival differed significantly according to functional impairment (ADLs: *χ*^2^ (2) = 39.0; *P* < .01; IADLs: *χ*^2^ (2) = 13.8; *P* < .01).

**Fig. 1:**
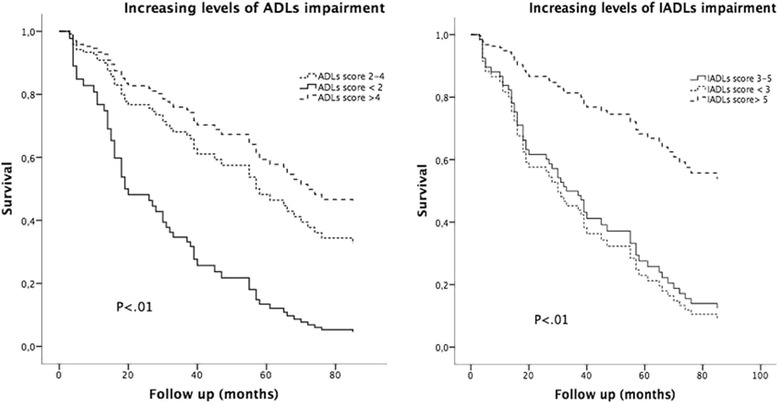
Survival curves according to the multivariable Cox regression model for increasing levels of ADLs and IADLs impairment. (Study center: Catholic University, Rome, 2007–2015)

According to the multivariable Cox regression model, survival was significantly reduced in patients with ADLs impairment; no significant differences in survival were detected among participants with IADLs impairment. In fact, according to multivariable Cox regression analysis (Fig. 1), impairment in the ADLs was associated with increased risk of mortality in crude model (HR 5.03; 95 % CI = 2.87–8.82), in a model adjusted for age and sex (HR 3.47; 95 % CI = 1.89–6.35) and in the fully adjusted model (HR 2.47; 95 % CI–1.07–5.67; Table 4), adjusted for those variables which showed significant differences in univariate analyses. In addition, similar results were observed after the exclusion of patients who died in the first six months of follow–up (crude model: HR 5.79; 95 % CI = 3.16–10.61; age and sex-adjusted: HR 3.85; 95 % CI = 2.00–7.40; fully adjusted HR 3.04; 95 % CI–1.26–7.38). The area under the ROC curve indicated that ADLs impariment was an useful predictor of death in all participants (0.72) and after the exclusion of subjects who died in the first six months of follow-up (0.71).

**Table 4:** Hazard ratio (HR) and 95 % confidence intervals (CI) of mortality at 90 months according to Cox regression models. All the covariates were entered simultaneously into the regression models. (Study center: Catholic University, Rome, 2007–2015)

	HR	95 % CI		HR	95 % CI
ADLs impairment	2.47	1.07–5.67	IADLs impairment	.80	.36–1.76
Age (years)	1.05	.99–1.10	Age (years)	1.06	1.01–1.12
Sex (female)	1.88	.93–3.81	Sex (female)	1.66	.83–3.33
Charlson comorbidity index	1.11	.92–1.33	Charlson comorbidity index	1.16	.96–1.39
Geriatric Depression Scale	1.04	.98–1.11	Geriatric Depression Scale	1.06	.99–1.13
MMSE^a^	1.04	.93–1.17	MMSE^a^	.99	.89–1.11
Serum creatinine (mg/dL)	.85	.73–.99	Serum creatinine (mg/dL)	.81	.69–.94
Serum albumin (g/dL)	.70	.21–2.27	Serum albumin (g/dL)	.72	.22–2.38

^a^ Mini Mental State Examination

A significant association between mortality and IADLs impairment was found only in the crude model (HR 2.79; 95 % CI = 1.58–4.91), but not in the age–and sex adjusted model (HR 1.59; 95 % CI = .85–2.96) nor in the fully adjusted model (HR .80; 95 % CI–.36–1.76, Table 4).

Similar results were observed after the exclusion of patients who died during the first six months of follow-up (crude model: HR 3.08; 95 % CI = 1.67–5.70; age and sex-adjusted: HR 1.76; 95 % CI = .90–3.44; fully adjusted HR .89; 95 % CI = .40–1.99). The area under the ROC curve confirmed that IADLs impariment was a poor predictor of death in all participants (0.66), as well as after excluding participants who died during the first six months of the follow-up (0.65).

In addition, according to Cox regression models, creatinine serum levels were associated with decreased mortality (model with ADLs impairment: HR .85; 95 % CI = .73–.99; model with IADLs impairment HR .81; 95 % CI = .69–.94), while age was associated with mortality only in the model that included IADLs impairment (HR 1.06; 95 % CI = 1.01–1.12). No significant association was found for the other covariates included in the adjusted Cox regression model. Also, the analysis of the interaction term indicated (*P*-.30) that the adjusted association between ADLs impairment and mortality did not vary according to creatinine serum levels. In addition, to avoid any possible inflated association of patients who died early (i.e. first six months of follow-up), the same cox model was run after the exclusion of these subjects.

Eventually, according to crude Cox regression, increasing impairment in the ADL score (P for trend < .01) as well as in the IADL score (P for trend < .01) was associated with reduced survival.

## Discussion

Results of the present study indicate that functional impairment is a highly prevalent condition in hemodialysis patients and that it is associated with reduced survival. Moreover, the presence of depression is associated with an increased probability of being disabled.

Little is known about the functional status of end stage renal disease patients receiving chronic hemodialysis. In fact, only few studies have investigated and defined the prevalence and the correlates of functional impairment in patients on chronic hemodialysis [6–9]. In keeping with previous studies which estimated a prevalent functional impairment ranging from 19 to 59 %, our data indicate a high prevalence of functional impairment in the ADLs, IADLs or both. Kurella Tamura et al. [6] have shown that the initiation of dialysis is associated with substantial and sustained decline of functional status in patients with end-stage renal disease who were living in nursing home; functional status was measured by assessing the degree of dependence in seven activities of daily living. Recently, Kavanagh et al. have shown that stroke, cognitive impairment, and higher systolic blood pressure were independent correlates of ADLs and IADLs impairment, while demographic characteristics, chronic health conditions, depressive symptoms or laboratory measurements were not [8]. The study of Kutner et al. demonstrated an increased probability of need for ADLs assistance in frail and prefrail hemodialysis patients, as well as in those with diabetes, lung disease, and stroke [9].

The results of the present study are intriguing, because depression might be a potentially reversible factor that might be easily and early identified after the onset of hemodialysis [17]. In fact, several previous studies confirm that serotonin-selective reuptake inhibitors may be safe also in patients with end-stage renal disease [17]. In addition, not-pharmacological treatments such as frequent hemodialysis, cognitive behavioral therapy, physical training programs, treatment of anxiety, and music therapy have led to promising results [17].

An association of ADLs and IADLs impairment with depressive symptoms or depression has been found in the general population, in older individuals, as well as and in patients with chronic diseases [18–24]. In older age, functional impairment and depression appear to coexist through a reciprocal relationship [18–24]. In fact, Bruce et al. found that depressive symptoms were associated with increased risk of functional impairment in community-dwelling elderly subjects [18]. However, participants were high-functioning men and women, and only ADLs impairment was assessed; on the contrary, our patients might be classified as “frail” patients. Also, Ormel et al. recognized three possible relationships between functional and depression, and concluded that functional impairment and depressive symptoms are mutually reinforcing over time [19]; however, their study included community dwelling elderly with significant initial physical limitations. Both ADLs and IADLs impairment were considered. In addition, Armenian et al. found an association between psychopathology and ADL impairment, only [20]; however, their study enrolled community dwelling participants, not only elderly, and major depression as well as other psychopathological conditions were taken into account. Barry et al. have identified an association between the severity of depressive symptoms and the burden of functional impairment [21]; the study included community dwelling elderly, and functional impairment was estimated only through ADLs.

Functional impairment may cause psychological distress, and depression may in turn lead to functional impairment through several mechanisms, such as reduction in motivation and help seeking, and changes in neural, hormonal and immunological systems which increase susceptibility to disease. Interestingly, a recent review has provided strong support for the bidirectional relationship between depression, functional ability and frailty in later life [24].

The present study also shows that impairment in the ADLs, but not IADLs, represents a risk factor for reduced survival in patients on chronic hemodialysis. These findings are consistent with previous observations that ADLs impairment is independently associated with higher mortality in patients on chronic hemodialysis [10]. It has also been demonstrated that the deterioration of even a single point of ADL on admission combined with age is highly predictive of poor outcomes in hospitalized dialysis patients [25]. Accordingly, functional impairment assessed by other tools has also been associated with higher mortality in patients on chronic hemodialysis [26, 27]. It has been proposed that rehabilitation through education, cognitive, physical, and psychosocial interventions might prevent, reverse or delay the onset of functional impairment with ensuing adverse outcomes in elderly dialysis patients, and thus should be incorporated routinely into standard dialysis care [28]. In our study, the absence of an association between mortality and functional impairment in the IADLs is noteworthy. Of notice, previous studies have indicated that impairment in the ADLs is a more sensitive risk factor for reduced survival in frailer patients [29, 30]. In fact, Mossakowska et al. found that ADLs, but not IADLs, predicted survival in a cohort of centenarians [29]. Delgado Parada et al. established that among patients hospitalized for heart failure the only variable independently associated with higher 1-year mortality risk was pre-admission dependence in performing the ADLs [30]. In addition, Sonmez et al. have ascertained that only the ADLs scale was a determinant of prognosis and survival in patients with breast cancer [31].

The evidence that higher serum creatinine levels were associated with increased survival and absence of functional impairment might deserve some concern. However, higher serum creatinine levels might reflect better nutritional status and higher lean body mass [32]; on the other hand, malnutrition and low muscle mass are acknowledged risk factors for both functional impairment and mortality [33, 34]. To confirm this hypothesis, in our population there was a significant positive correlation between serum albumin and creatinine levels. Indeed, no correlation between creatinine levels and body mass index was found; however, the body mass index is not a good indicator of muscle mass [35].

The present study has some limitations. First, the sample size was relatively small, which may make any generalization difficult. This limitation, which is reflected by the width of some confidence intervals, also restricted the number of covariates that could be entered into the regression models. Second, we don’t know the dynamic nature of functional impairment, and consequently we may have included both patients with transient functional impairment and those with chronic, persistent functional impairment. However, it is well known that self-reported functional ability is valid in non-dialysis populations and it has been shown to be useful and valid also in patients on chronic hemodialysis [9, 10].

## Conclusion

The present study shows that impairment in ADLs and IADLs is a common finding, independently associated with depressive symptoms. ADL impairment represents a risk factor for reduced survival in patients on chronic hemodialysis. Further studies are needed to better understand the mechanisms underlying such associations and to identify subjects who might benefit from interventions aimed at improving survival.
